# Pike-Perch (*Sander lucioperca*) and Rainbow Trout (*Oncorhynchus mykiss*) Fed with an Alternative Microorganism Mix for Reducing Fish Meal and Oil—Fishes’ Growth Performances and Quality Traits

**DOI:** 10.3390/foods10081799

**Published:** 2021-08-04

**Authors:** Michaela Schafberg, Karin Loest, Andreas Müller-Belecke, Sascha Rohn

**Affiliations:** 1 Institute of Food Chemistry, Hamburg School of Food Science, University of Hamburg, Grindelallee 117, 20146 Hamburg, Germany; schafberg@chemie.uni-hamburg.de; 2 IGV Institut für Getreideverarbeitung GmbH, Arthur-Scheunert-Allee 40/41, 14558 Nuthetal, Germany; karin.loest@igv-gmbh.de; 3 Institute of Inland Fisheries Potsdam-Sacrow (IfB), Im Königswald 2, 14469 Potsdam, Germany; andreas.mueller-belecke@ifb-potsdam.de; 4 Institute for Food and Environmental Research (ILU) e. V., Papendorfer Weg 3, 14806 Bad Belzig, Germany; 5 Department of Food Chemistry and Analysis, Institute of Food Technology and Food Chemistry, Technische Universität Berlin, TIB 4/3-1, Gustav-Meyer-Allee 25, 13355 Berlin, Germany

**Keywords:** fish meal substitution, rainbow trout (*Oncorhynchus mykiss*), pike-perch (*Sander lucioperca*), *Arthrospira* sp., *Crypthecodinium cohnii*, *Rhodotorula glutinis*, sensory impression

## Abstract

In the last decades, several plant-based materials were used for the substitution of fish meal and oil in aquaculture. The present study evaluated the fish quality and the sensory differences of rainbow trout (*Oncorhynchus mykiss*) and pike-perch (*Sander lucioperca*) from three different feeding groups, which were fed a commercially available industrial (standard) diet, a control diet, and a special microorganism-based feed mix. This feed mainly consisted of a mix made of *Rhodotorula glutinis, Crypthecodinium cohnii,* and *Arthrospira* sp. and had 50% less fish meal and fish oil compared to typical control diets. At the beginning, the pike-perch population was six months old, and the rainbow trout population was 15 months old. The feeding study duration was 16 weeks and every four weeks the growth performance and several morphometric parameters were recorded. Afterwards, sensory evaluation took place to identify possible trends. Sensory evaluation revealed that the rainbow trout groups did not show any significant differences to the standard and control fish fillets with regard to odor, texture, and taste. The effects on rainbow trout growth performances and carcass parameters were similar to the standard group. The feed mix was not optimal for pike-perch farming, which was also reflected by significantly adversely affected growth performance and carcass parameters. The sensorial evaluation showed an opposite trend: here, only small differences in the fillets from the feed mix and standard/control diet were observed.

## 1. Introduction

Fisheries, aquaculture, fish consumption, and their perception have already changed a lot in the last decades [1,2,3]. One of the consequences of oceans’ overfishing was, in addition to the increasing aquaculture sector, the protection of livestock, regulated by EU guidelines [4,5,6]. Additionally, this was supported by the intensified medial spread of the health-beneficial aspects of fish consumption [2,7], as well as pressure from animal protection associations [8,9]. Fish is recommended as being an important part of a healthy diet for humans, because of its high-quality protein, its fatty acid profile, and its micronutrients. It is also low in carbohydrates, saturated fatty acids, and cholesterol [2,3,10,11].

Two important, but very controversially discussed fish feed ingredients are fish meal and fish oil. Fish meal and fish oil production is a huge industry that forces the exploitation of local seas and, in most cases, it is not sustainable at all, although a quarter of the world’s fish meal is made of fish processing waste as a raw material [3,11,12,13,14,15]. Another imported aspect is the price for fish meal and oil, which has significantly increased in the last decades [13].

Even when the content of fish meal and oil is continually reduced, both ingredients were regarded as the simplest source of proteins and polyunsaturated fatty acids (PUFA), such as omega-3 or omega-6 fatty acids [16,17]. Moreover, as an additive or ingredient, fish oil has a huge impact on the taste and flavor of the final fish products [18,19]. However, this needs to be addressed and alternatives need to be developed. So far, fish meal and oil have been partially or completely substituted in many fish feeds by plant-based materials, but also more innovative biomasses such as microorganisms (algae/cyanobacteria) [20,21]. In addition, plant-based oils or concentrates made from, e.g., soy [22] and other vegetables, have been used [23,24,25]. The listing of these examples is not claiming to be complete, but it should illustrate the intense variation and efforts that have been conducted in the recent years.

When substituting fish oil, special attention has to be given to the PUFA content, as these are indispensable for humans. They are not or insufficiently biosynthesized in humans and should be supplied by eating marine fish. Consequently, PUFA content in fish should not be diminished by alternative feeds [26,27]. Zhu et al. (2021) compared the replacement of fish oil (0%, 50%, 100%) with microalgae (*Aurantiochytrium*) or astaxanthin in young rainbow trout and found that the replacement of 50% fish oil had no negative effects on fishes’ growth performance [28]. For example, Øverland et al. (2009) substituted fish meal with pea protein in diets for Atlantic salmon (*Salmo salar*) [29], whereas Barrows and Frost [30] used co-products from the nut industry, algae, and invertebrate meals in diets for rainbow trout (*Oncorhynchus mykiss*) [30]. The sensory aspects such as the variation of the fillet color or the skin color are typical parameters that need to be considered, as well [13,31]. However, the variations of taste, flavor, and texture of the fillets, especially, of rainbow trout (*Oncorhynchus mykiss*) and pike-perch *(Sander lucioperca*) were hardly taken into account and accordingly have not been described yet comprehensively.

In 2019, around 18,500 t of fish and fishery products were produced in German aquaculture, a third came from rainbow trout (~6200 t) and 57 t were pike-perch [32]. Furthermore, these two species represent typical edible fish in Germany. They are low-PUFA freshwater fishes and important for human nutrition. Schafberg et al. (2020) already showed in a recent study the effects of an innovative feed on antioxidant activity with regard to the course from raw materials over feed production into the fillets of rainbow trout [31]. In that study, three diets were compared: a commercially available industrial (standard) diet, a control diet, and a new feed mix. In that mix, fish meal and oil content were reduced by half (compared to the control diet). For preventing nutrient deficiency, a mixture of the microorganisms *Crypthecodinium cohnii, Arthrospira* sp., and *Rhodotorula glutinis* was used [13,31]. *R. glutinis* was chosen as the new lipid source. *C. cohnii* was used to increase the PUFA content, in particular the docosahexaenoic acid (DHA) content, because *C. cohnii* can contain up to 50% DHA per dry matter [18,33,34]. Instead of fish meal, *Arthrospira* sp. was used as the new additional source of proteins [13,18,31]. In order to ensure sustainable aquaculture, microorganisms were cultivated in biotechnological processes [18,33,34]. Schafberg et al. (2020) showed that the microorganism-based feed mix led to healthy fish, which were comparable to conventionally fed fishes and led to the accumulation of carotenoids and other antioxidants in the fish fillets [31].

The present study aimed at evaluating the growth and quality traits of rainbow trout (*Oncorhynchus mykiss*) and pike-perch *(Sander lucioperca*). Both are fish being typically consumed in Germany. Further, they were chosen for comparing between a predatory and non-predatory species. It was hypothesized that predatory fishes cannot cope well with that new fish feed mix. As growth and health performance parameters, weight, length, specific growth rate, feed conversion ratio and k-factor were chosen. Liver color was used to reveal possible adverse effects in liver metabolism and lipid accumulation. Besides, carcass parameters such as length, weight, and color are important for fish farmers, but these are also very important decision criteria for consumers. For them, the fillet of the fish is the most valuable part due to several factors, such as health benefits, taste, appearance, or growing conditions (conventional vs. (organic) sustainable aquaculture) [35,36,37,38,39]. Consequently, the sensory evaluation was regarded as a further important aspect, and the color, odor, texture, and taste of the fish fillet were also characterized in the present study. The accumulation of any substances with positive health benefits is irrelevant when the product does not meet consumers’ expectations and acceptance.

## 2. Materials and Methods

### 2.1. Feeds

Feed mixes and their nutritional parameters were already described by Schafberg et al. [31]. The commercially available industrial (standard) feed (ALLER METABOLICA, 4.5 mm; Prod. No. 41400342) was purchased from Alleraqua GmbH, Golssen, Germany. IGV GmbH (Nuthetal, Germany). The control feed was created and produced by the very well-experienced industry partner *vitafeed* Spezialfuttermittelwerk Beeskow GmbH (Sfw; Beeskow; Germany), based on a well-established recipe with 37.9% fish meal and 13.6% fish oil. The new feed mix was based on the same recipe as the control diet, but the feed mix consisted only of half of fish meal and fish oil. The other half was replaced by a mix of *Arthrospira* sp. (12.1%)*, Crypthecodinium cohnii* (4.5%), and *Rhodotorula glutinis* (9.0%). The pellets had a diameter of 4 mm and were stored at room temperature until feeding.

### 2.2. Fish Feeding Trials

#### 2.2.1. Experimental Set-Up

The fish feeding trials took place at the Institute of Inland Fisheries Potsdam-Sacrow (IfB), Germany. The trials took 16 weeks (from the end of July 2015 to mid-November 2015) and three different diets (S: commercially available industrial (standard) diet; C: control diet; FM: feed mix) were tested with pike-perch (406 individuals in total) and rainbow trout (391 individuals in total) in duplicate. The trials were performed under aquaculture conditions of a semi-industrial scale. Pike-perch was kept in a recirculation aquaculture system including six rearing tanks of each 1.4 m^3^. The water temperature was 22 °C. Rainbow trout were kept in a recirculation system at 11 °C, using deep water of the adjacent Lake Sacrow (Potsdam-Sacrow, Germany) for cooling by heat exchangers. Six groups were kept per experiment (two groups per diet). Both species were initially stocked into the tanks for acclimation for two weeks. During the first four weeks of the feeding studies, the maximum daily amount of consumed feed (FI max.) was determined. In the further course, to achieve optimal feed conversion, the daily feed administration was set about 10% below the maximum feed amount. The percentage of these amounts was therefore based on the fish biomass of each group and the species available at the start of the experiment and was adjusted monthly after each weighing. The populations were automatically fed every four hours for the whole trial period. Consequently, slowly growing groups therefore received less feed in total. This approach is unavoidable, as non-consumed feed can lead to massive pollution of the water and the fish. The fish harvesting was based on the codes of good practice for food-producing fish for human consumption.

#### 2.2.2. Fish Samples

The IfB cultivated their own pike-perch fingerlings, which were approx. 6 months old. The tested pike-perch population consisted of 66 to 70 individuals (average initial weight 174 g ± 44 g) per feeding group (S; C; FM) and tank (two tanks per diet). The rainbow trout population was obtained from a culture from Saxony-Anhalt (Germany) and the fish were approx. 15 months old. The tested rainbow trout population consisted of 64 to 66 individuals per diet (S; C; FM) and tank, with an approx. weight of 139 g ± 33 g.

The fish of each tank were weighed and measured once a month (pike-perch: day 0, 35, 63, 91, and 120; rainbow trout: day 0, 31, 59, 89, and 115). At the end of the trials, fish were removed from the tanks, anesthetized, and killed in accordance with the German Animal Welfare Act. The morphometric traits, growth and other parameter were analyzed directly after the killing. Afterwards, the organs were removed, and the fish was filleted. All samples were collected in plastic bags and stored at −20 °C until analysis. The fish fillets were pooled for the analysis of the proximate composition and the metabolites.

#### 2.2.3. Growth Performance and Carcass Parameter

The following growth performance and carcass parameters were estimated for all three diets at the end of the trial: Weight gain [%], total fish length [cm], fillet weight [g], liver weight [g], intestinal fat [g], and survival rate [%]. The specific growth rate (SGR) was calculated as (log_e_ weight _end_ [g] − log_e_ weight _start_ [g])/days × 100 according to Busacker et al. [40]. The condition factor (k-factor) was calculated as weight [g]/lenght^3^ [cm^3^] × 100 according to Fulton (1904) and the feed conversion ratio (FCR) as the amount feed [kg]/increased weight [g] [41]. The intestinal fat somatic index (IFSI) is the ratio of intestinal fat weight [g] to fish weight [g], whereas the hepatosomatic index (HSI) describes the ratio of liver weight [g] to fish weight [g]. The survival rate was calculated over the whole study period. The colors of the fresh (as well as lyophilized) fillet and the fresh (as well as lyophilized) liver were described using CIE color space L*a*b* [42,43,44]; this is indicated by the luminosity (L), level of redness or the red–green axis (a), and yellowness level or the blue–yellow axis (b). The color difference (dE) is explained in more detail. On the one hand, the color difference of the liver (dE_week4_) between Week 4 and Week 16 was compared and, on the other hand, the color difference (dE_Feedmix_) between the liver color of the diet FM to S and C was compared after 16 weeks. When the dE value is more than 1, it is defined as distinguishable to the practiced eye.

### 2.3. Chemical Parameters

#### 2.3.1. Chemicals

Acetyl chloride, n-heptane, disodium hydrogen phosphate, methanol, sodium chloride, and sodium sulfate were purchased from Sigma-Aldrich Chemie GmbH (Taufkirchen, Germany). Sodium carbonate was purchased from Grüssing GmbH (Filsum, Germany). All chemicals used in the experiments were of analytical grade and all aqueous solutions were prepared with ultrapure deionized water.

#### 2.3.2. Fillet and Liver Color

The determination of the fillet and liver color was analyzed photometrically according to Schafberg et al. and the definite color was determined using the CIE-Lab color space [31].

#### 2.3.3. Determination of the Fatty Acid Profile

According to Schafberg et al. (2018), the fatty acid (FA) profiles of the fish fillets were determined as fatty acid methyl esters [13]. The results were expressed as g fatty acid 100 g^−1^ biomass. The limit of quantification (LOQ) was 0.005 g FA 100 g^−1^ weight.

#### 2.3.4. Determination of Ash, Dry Mass, and Protein Content

The ash, dry mass, and protein content of the fish fillets were analyzed according to Schafberg et al. [31]. The results were expressed as g 100 g^−1^ BM. The LOQ for all three methods was 0.01 as g 100 g^−1^ BM.

#### 2.3.5. Statistical Analysis

All analyses were done in triplicate for the statistical analysis. For the identification of significant differences between the groups C, FM, and S, all data were analyzed by descriptive statistics and paired sample *t*-tests using IBM^®^ SPSS Statistic version 26. Levene’s test was used to check for homogeneity of variance. The significance level was set at *p* < 0.05 and the values were expressed as the mean ± standard deviation (SD). In the corresponding tables, the letters “F” and “K” explain the significant difference. Moreover, as “C” is used as an abbreviation for the control groups, it was tried to avoid misunderstandings. “F” is the significant difference between the standard (or the control) to the feed mix. “K” is the significant difference between the standard and the control.

### 2.4. Sensory Evaluation

#### 2.4.1. Paired Comparison Test

The sensory evaluation took place at the partner site IfB from 11 to 22 December 2015. The tests were done in paired comparison tests according to ISO EN 5495. The thawed fillet samples were heated in the oven at 180 °C for 10 min and presented to the panelists. The sensory panel consisted of six experienced panelists and the study was divided in three parts (Test I: commercially available industrial (standard) diet vs. new feed mix, Test II: control diet vs. new feed mix, and Test III: standard industrial diet vs. control diet) per species. Each part was run four times to ensure the required sample number (*n* = 24). The paired comparison test focused on the intensity/expression and preference of the following characteristics: odor, color, texture, and taste. As an example, when comparing the two fillets (A vs. B), the panelists should decide whether the respective aspect (here: odor) of the two fillets differed. If there was a difference, it was checked which of the two fillets had a more intense odor and which the panelist would prefer. The possible answer options were ‘no difference’ (ND), A/B more intensive, A/B preferred. In addition to those aspects, experts were asked to write down characteristics that attracted their attention. The panelists were free to choose words for their impressions; no guidelines were given here.

#### 2.4.2. Statistical Analysis

No-difference-decisions by the panelists were assigned to both products tested in equal parts. The default procedures for statistical analysis of the international ISO EN 5495 norm for both-sided tests were then followed. The significance level was set at *p* < 0.05.

## 3. Results and Discussion

Over the last decades, many studies tested the effects of the substitution of fish meal and oil in fish feeds used in different kinds of aquaculture. For a partial substitution, several plant-based materials such as nut meal [30] or concentrates of pea protein, horse bean protein, and rapeseed protein [45] have been used. In addition, a wide variety of microorganisms, especially single-cell organisms, are used in the present aquaculture and their cultivation has been optimized accordingly over the last decades [27,46,47]. Further, microorganisms, e.g., up to 10% *Spirulina platensis*, have also been used for rainbow trout feeding [20,48,49]. However, only a few studies that used microorganisms in their feed mixes analyzed the sensory attributes [50], besides the quality traits and growth evaluation of the final fish or fish products. For instance, Schafberg et al. (2018) already described the positive effects of a microorganism mix (*Rhodotorula glutinis, Crypthecodinium cohnii, Arthrospira* sp.) as a feed ingredient on the compatibility of rainbow trout and pike-perch [13]. Afterwards, Schafberg et al. (2020) used a different composition of the microorganism mix and described stabilizing effects on the antioxidant profile during the manufacturing process [31]. The present study used the same mix as described by Schafberg et al. [31], but focused on the growth performance, feed conversion, carcass parameters, and especially, the sensory description and evaluation of the fish fillets.

### 3.1. Fish Growth Performance and Morphmetric Traits

#### 3.1.1. Pike-Perch

The present study underlined the results described by Schafberg et al. (2018) for a pike-perch population [13]. Pike-perch as a predator was forced to cope with this new, comparatively more vegetarian diet, as 50% of the fish meal and oil were substituted. Pike-perch and rainbow trout are both carnivores, but the pike-perch farming is a bit more challenging, as rainbow trout can cope with a higher proportion of vegetarian ingredients. The growth performance and feed conversion in this study showed that the FM acceptance and conversion was not adequate for pike-perch (Table 1). An important reason for this could be that the digestion of the microorganisms’ biomass, especially their cell walls, is insufficient. Carnivores differ in the digestion/adsorption process of complex carbohydrates compared to herbivores and omnivores in the conversion rates. Carnivores have, e.g., lower pancreatic and intestinal enzyme activity towards complex carbohydrates. So, this can also lead to a slower and insufficient conversion of complex polysaccharides and following this also lower growth rates and weight gain [51,52,53].

Pike-perch showed significant differences in the final fish weight, but also already in the weight gain. The standard diet led to a weight gain of 278 g ± 102 g, compared to the initial weight. So, the fish had grown by about a factor of 2.6. Fish fed with Diet C had a weight of 217 ± 67 g, which means that the fish initially gained weight by a factor of about 2.2. Fish fed with Diet FM gained “only” twice of their initial weight (183 g ± 74 g).

The fillets resulting from group FM were significantly smaller (144 g) than the fillets of the feeding group S (185 g), but there were no differences to fillets from Group C (165 g). Generally, the fillet made up about approximately 40% of the total weight for each group.

The intestinal fat and the IFSI showed significant differences. Fish from Group S had 28 g fat (IFSI: 6.3%), whereas Diet FM led to less than half of the intestinal fat (12 g fat; IFSI: 3.4%), and Diet C led to 18 g fat (IFSI: 4.5%). The livers of the Diet S fish weighed significantly more (5.4 g) than the livers of the fish that were fed with Diets C (4.3 g) or FM (4.3 g). These values were expected due to the larger increase in the mass of the S-fish compared to the other diet groups.

The SGR of the fish fed with Diets FM or C (0.6% day^−1^) were lower than those resulting from Diet S (0.8% day^−1^). The same trend was shown at the hand of the k-factor (S: 1.0, FM: 0.8, and C: 0.9). The feed conversion ratio (FCR) of Diets S and C (both approx. 1.1) were significantly lower than of Diet FM (1.6). Regardless of the diet, the fish showed no significant differences in their survival rate (99%), total length (~35 cm), and HSI (~1.2%). On the one hand, the HSI values (~1.16%) in the present study were slightly higher than Lazo et al. (2017) reported for pike-perch (0.8%; *n* = 10). On the other hand, the sample size in the present study was also higher (*n* = 66 and 68). The present HSI results were still in a range that was comparable to values for farmed fish [54].

Liver color was used to reveal possible adverse effects in liver metabolism and fat accumulation (Table 2). A shift in luminosity (L) and/or an increase in the yellowness level (b) can indicate a possible fatty liver. Even though the liver color in Week 16 did not show any significant differences, the calculated color difference compared to Week 4 showed that all three parameters increased. The liver color resulting from Diet FM showed the lowest values overall (e.g., L = 42.1), which resulted in the high color differences compared to the liver color of Groups S and C. From this, it can be concluded that there was no increased lipid accumulation in the liver in FM-fed pike-perch, being a kind of evidence of healthy individuals. This is an important part of excluding non-alcoholic fatty liver disease (NAFLD), because it is the most common cause of chronic liver disease for all species. It is a clinical syndrome characterized by predominant macrovesicular steatosis of the liver [55]. In the case of disturbed lipid digestion, changes in diet and lifestyle can lead to an improvement in health. Therefore, attention should be paid to the components contained in the food, as these have a direct impact on health or can promote diseases [55,56].

On the basis of the parameters mentioned so far, comparably healthy individuals of all diet groups were assumed, so the (quality) parameters of the fillets were then determined, since it is the most important part of the fish for the consumer. In addition to the fillet color (Table 2), the total lipid content as well as the protein content or the water content are important here (Table 3). The fillets of the FM group had significantly lower amounts of proteins (FM: 20.5%, C: 20.3%, S: 21.2%) and dry mass (FM: 23.6%, C: 24.4%, S: 23.9%) but no significant differences in their water content and total lipid content. With regard to the feeds, there were no significant differences in their dry mass, ash, and total lipid content, but the FM diet had lower amounts of theses nutrients than the other diets [31]. Moreover, the protein contents in the diets did not show significant differences (FM: 46.3%, C: 45.8%, S: 45.8%) [31]. The distribution of nutrients in the feed cannot be transferred 1:1 to the individuals, which shows how the metabolism varies due to the bioavailability of the ingredients.

The fillets of the FM group had the brightest color (L = 44.7), the lowest redness level (a = 1.08), and a higher yellowness level (b = −1.58) than fillets from Groups S and C (Table 2). The fillets of Groups S and C fish differed significantly from the FM fillets. Those parameters led to dE values >1 compared to FM, but it could be already determined visually that the FM fillets were slightly brighter. The lower redness level means a color shift into green compared to the C and S fillets. The reason for the “green shift” is the accumulation of chlorophylls from *Arthrospira* sp., whereas the higher yellowness level resulted from the increased carotenoid content of the microorganisms in the FM diet [13,20,31].

In the present study, all feeding groups showed no differences in the total lipid content between each of the pike-perch fillets, ~1% being a typical value for such a lean fish. In this context, Lazo et al. (2017) already showed similar values in the dry mass, ash, and protein content of pike-perch samples, but a comparatively lower total lipid content [54].

A closer look at the fatty acid profile showed that the fillets of the fish fed in Groups S and C had the following fatty acids predominantly in the fillets, in descending order: oleic acid (18:1), palmitic acid (16:0), linoleic acid (18:2n6, LA), stearic acid (18:0), and DHA (22:6n3). The FA profile in the FM fillets was slightly different, and oleic acid and palmitic acid were also the two dominant FA, but the oleic acid content (30.0% fat^−1^) was significantly lower than in the S fillets (34.6% fat^−1^), and the palmitic acid (29.1% fat^−1^) was significantly higher in the FM fillets than in the S fillets (23.6% fat^−1^). The following FA in the FM fillets were stearic acid (7.27% fat^−1^), LA (6.64% fat^−1^), and palmitoleic acid (6.36% fat^−1^). All three diets showed the same three predominant fatty acids in descending order: oleic acid, LA, and palmitic acid [31]. The amount of oleic acid in the S diet (50.1% fat^−1^) was significantly higher than in the FM (30.5% fat^−1^) or C diet (32.3% fat^−1^) [31]. Moreover, the FM and C diets showed significantly higher DHA (~4.6%) and EPA (FM: 2.3% fat^−1^, C: 3.0% fat^−1^) contents than S (DHA: 1,2% fat^−1^, EPA: 1.3% fat^−1^) [31]. The high content of oleic acid, palmitic acid, stearic acid, and LA in the fillets goes hand in hand with the high content of the same fatty acids in the diets [31]. Moreover, the high content of oleic acid and palmitic acid in all fillet groups is in agreement with the findings of previous studies about fatty acids in pike-perch, where oleic acid and palmitic acid were also reported as the dominant fatty acids in farmed and wild pike-perch [13,57,58].

PUFA content in pike-perch fillets consisted mainly of LA, DHA, and eicosapentaenoic acid (20:5n3; EPA). These were significantly higher in the S and C fillets than in the FM fillets.

The feeding period of the present study was twice as long as the previous study described by Schafberg et al. [13]. Consequently, the present individuals were also larger and heavier. This goes along with the higher total lipid content of the fish. In relation to the biomass, the present study showed higher PUFA and lower MUFA (monounsaturated FA) contents than described by Schafberg et al. [13].

Thus, the changes in the FM recipe led to a good PUFA enrichment in the pike-perch fillets in the present study. The accumulation of palmitoleic acid (16:1n7) in FM fillets (6.36% fat^−1^) was significantly higher than in the fillets from Groups S (0.91% fat^−1^) or C (0.77% fat^−1^). Palmitoleic acid is metabolized from palmitic acid via the stearoyl-CoA desaturase pathway [13,59,60,61,62], this pathway seems to be favored by the intake of the FM diet.

Schafberg et al. [31] showed that the standard feed had a significantly higher palmitic (2.22 g FA 100 g^−1^) and palmitoleic acid (0.30 g FA 100 g^−1^) content than the FM diet (palmitic acid 1.58 g FA 100 g^−1^, palmitoleic acid 0.03 g FA 100 g^−1^) and the control feed (palmitic acid 1.88 g FA 100 g^−1^, palmitoleic acid 0.03 g FA 100 g^−1^). However, when these fatty acid contents were related to the total lipid content, there were no significant differences between the S (palmitic acid 23.6% fat^−1^, palmitoleic acid 0.91% fat^−1^) and FM diets (palmitic acid 29.1% fat^−1^, palmitoleic acid 6.36% fat^−1^), whereas Diet C showed a slightly lower content of those acids (palmitic acid 23.1% fat^−1^, palmitoleic acid 0.77% fat^−1^). Therefore, the significantly higher content of palmitoleic acid in the fat of the FM fillet must result from the microorganisms’ content of the FM feed. So, the reaction between stearoyl-CoA desaturase and palmitic acid to form palmitoleic acid seems to be favored by the content of microorganisms in the diet.

The FM fillets had significantly lower DHA, EPA, and arachidonic acid contents than the fillets of the S and C groups, although the FM diet had higher DHA and EPA contents than the S diet [31]. The FM diet seems to influence the fatty acid metabolism of the pike-perch in that the elongation of the fatty acids is reduced. As a freshwater fish, pike-perch tends to metabolize LA and α-linolenic acid (ALA). Thus, at the beginning of the elongation cascade, LA and ALA are competitive substrates for the existing desaturases and elongases in fish in order to form very-long-chain PUFA and to build them into lipids and membranes [60,61,62].

The significantly lower DHA content in the FM fillets underlined that a longer feeding trial was not necessarily an approach for a better accumulation. Another important point for the low DHA content is that the diets (FM, S, and C) were not optimized or adapted for pike-perch. This study was an attempt to see whether pike-perch would accept this new diet formulation or reject it in general. Similarly, as described by Schafberg et al. (2020), the rainbow trout fillets had higher DHA contents in their fillets than the pike-perch fillets [31]. Both species received the same feeds, so the low DHA content must result from their species-specific metabolism.

The discussion about the FA profile in the context of other pike-perch studies should be done carefully. The ‘physiology’ and the biosynthesis of specific metabolites such as fatty acids depend above all on the species, and study design (age, size, sex, season, marine or freshwater fish). In addition, geographical location and water temperature for fish are decisive for which FAs are metabolized [58].

#### 3.1.2. Rainbow Trout

The innovative microorganism-based feed mix of the present study was especially optimized for the rainbow trout population, based on previous results [31]. Additionally, Pike-perch was chosen for comparing between a predatory and non-predatory species. It was hypothesized that the non-predatory rainbow trout will cope more easily with that new fish feed mix.

Table 1 shows the growth performance and feed conversion parameters. The fish did not show any significant differences in the initial weight (139 g ± 33 g), but at the end of the study, FM led to a significantly lower weight (410 g) than the diets of the two other groups. The same trend was shown at the hand of the weight gain, the fillet weight, the total length, the liver weight, k-factor, SGR, as well as the intestinal fat, the IFSI, HSI and the FCR. The fish did not show significant differences depending on the diet. In the present study, only 50% of the fish meal and oil content were substituted. Previous studies showed that plant-based feeds show reduced growth performance (especially low weight gain and high FCR), when large portions of fish meal or oil were replaced [45,63,64,65,66]. In the present study, the control diet consisted of ~50% fish meal and oil. For FM, the half of these ingredients was substituted. In the present study, FCR was at the same level for all feeding groups, whereas the weight gain differed significantly. This result was also shown in previous studies [63,64]. The substituted part is therefore a decisive criterion. The aim of the present study was to create a feed that provides a similar growth performance to commercially available feeds (with traditional portions of fish meal and oil). The SGR of the rainbow trout fed with FM was slightly lower than for fish of Groups C and S, but the difference was not significant.

Fingerlings gain more weight in a shorter time than older individuals, because of their developmental stage of growth. In the present study, older fish were used over a comparatively longer study period and showed, expectedly, a lower weight gain. However, the trend is the same; the ratio of FM to C was 0.73 in the study mentioned before [30], whereas in the present study, the ratio was 0.67 for FM to C and 0.60 for FM to S.

The survival rate was slightly lower compared to the previous study described by Schafberg et al. [13]. The growth performance and feed conversion confirmed that FM was well adapted by rainbow trout, but still bearing a potential in terms of weight gain and final market weight. The added value should be in the focus and sustainable practices should also be a priority, like in other sustainable agricultures.

After 16 weeks, the liver color of the fish of the feeding group C was generally darker than those of S and FM fish (Table 2). There were no differences in redness levels, but the yellowness levels of S and FM livers were higher than those of C livers. The high dE_feedmix_ of C livers resulted in a shift from yellow to blue. The color difference dE_week4_ resulted from various changes: the yellowness level in the S livers increased, while it was decreased in the C and FM livers. The decrease in lightness of the liver color also contributed to the increased dE value of the C livers. From the color of the liver, conclusions can be drawn about the accumulation of fat. The results show that the FM diet resulted in individuals that were as healthy as the individuals of the standard diet group, as the dE_feedmix_ of S-fed livers was relatively low and the dE_feedmix_ was about half compared with fish fed with feed C. This means that the liver of FM fish looks more like the liver of S fish than the liver of C fish.

Schafberg et al. (2020) already showed the results of the nutritional parameters of the fillets (Table 3) and the feeds, accordingly [31]. The dry mass, ash and protein content of the fillets did not show significant differences [31]. There were significant differences in the fat content (FM: 7.5%; C: 7.8%; S: 8.2%) and in the content of some PUFA in the fillets, more precisely ALA, EPA, and DHA (Table 3) [31]. The four predominant fatty acids (in descending order) in the fillets were: palmitic acid, LA, stearic acid, and oleic acid. These fatty acids were also dominant in the feeds, as described above (3.1.1.) [31].

Authors further described the color of the lyophilized fillets [31], whereas the present study focused on the color of fresh fillets. The fillet color of the fresh FM fish (Table 2) was significantly different (L = 41) and not as bright as the color of the fresh fish resulting from Diets S (L = 43) or C (L = 43). However, fresh FM fillets had the highest redness and yellowness level. These differences also explain the high dE values. The same trend was observed in the lyophilized samples [31]. The fresh and lyophilized samples of S and C fillets were slightly brighter than the FM fillets, with the lightness of fresh fillets (L = 42) being about half of the lyophilized samples (L = 72). The redness level of the fresh C (a = 1.5) and S fillets (a = 2.0) was significantly lower than in the FM fillets (a = 2.6), whereas the lyophilized fillets of C (a = 1.8) showed the highest redness level (FM_lyo_: 1.1; S_lyo_: 0.2). Moreover, as expected, the yellowness levels of the lyophilized samples were significantly higher (b = 20.2) than in the fresh samples (b = 3.5). The differences in the yellowness level can unexpectedly be observed more clearly in the fresh fillets (C: 0.63; FM: 6.94; S: 3.01). As already described for pike-perch, a lower redness level means a color shift towards a green color. The reason for such a “green shift” is the accumulation of chlorophylls from the ingredient *Arthrospira* sp., whereas a higher yellowness level results from increased carotenoid contents [13,20,31]. These results confirm outcomes of previous studies, where fish meal was substituted with *Spirulina platensis*. Teimouri et al. (2016) showed that all fillets that were fed with spirulina had a significantly lower luminosity, higher redness, and higher yellowness levels than the control fillets [20]. Even more, the increasing amounts of spirulina in the feeds correlated with an increasing redness and yellowness level and the decreasing luminosity [20]. Therefore, the change in the FM fillet colors in the present study can be assigned to the spirulina content in the feed.

### 3.2. Sensory Evaluation

**Figure 1:**
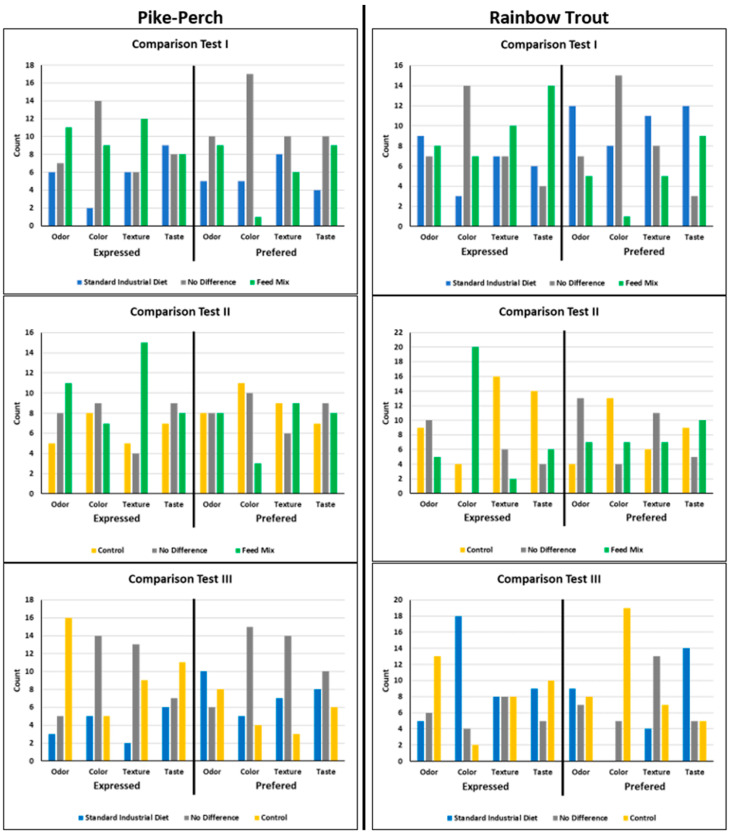
Comparison Test I-III of pike-perch (**left**) and rainbow trout (**right**). The left side of each sub-figure (**I**–**III**) shows the intensity distribution of the “expressed/distinct” attribute (taste, odor, texture, and color), and on the right side the “preferred” attributes of the sensory test are shown. Comparison Test I: comparison of the standard feed (S; blue) with the feed mix (FM; green), Comparison Test II: FM vs. the control feed (C; yellow), and Comparison Test III: S vs. C. When there was no significant difference between the respective aspects, ‘no difference’ (ND, grey) was noted accordingly.

In order to perceive sensory differences more clearly, three comparison tests were carried out (Figure 1). Here, fillets from two different diets were compared with each other (Test I: FM vs. S; Test II: FM vs. C; Test III: C vs. S). In this study, the standard diet represented a typical, commercially available fish feed, which is used for conventional fish farming. Consequently, consumers are used to accepting the resulting fish fillets with regard to color, odor, taste, and texture. The control feed and the feed mix were based on an almost similar recipe: C consisted of ~50% fish meal and oil, whereas FM contained only half of that. All fish groups were farmed under the same conditions. Consequently, the sensorial changes can only refer to the feeds, but not environmental factors. Feed C differed from FM in the content of fish meal, fish oil, and the further constituents of the microorganism mix. Sensorial differences between the control fillets and the FM fillets can only be due to the microorganism mix. Other important aspects are the strength of the sensory attributes and how they affect potential consumers (‘like or dislike’). Therefore, another aspect was that the panelists had to differentiate between more intense and preferred attributes.

#### 3.2.1. Pike-Perch

The three paired comparison tests of pike-perch fillets (Figure 1, Table S1) showed that the FM fillets had differences in the intensities of the odor and the texture, but the preferences were more balanced. The only significant difference was the intense odor of the control fillets compared to the standard fillets (Test III), while there was no preference in the smell. However, panelists made the most comments on the odor (Table 4). The C and FM fillets were also described as ‘with a foreign smell’ or ‘more intense’ compared to the S fillets. Lazo et al. (2017) described pike-perch as ‘earthy’ or ‘musty’ in flavor. However, this impression was not the dominant attribute by any feeding group of the present study [54].

Fillets of lean fish have less fat and a higher moisture content, also resulting in different textures [67,68]. As described above, the total lipid contents showed no significant variations, but the moisture of FM fillets was significantly higher than for the S and C fillets. Panelists’ comments on the texture were balanced and no group was preferred. Even though the colors of the fillets had no significant differences in the compared test, the panelists described the FM fillets as ‘darker’ than the other groups. Correspondingly, fillets of S and C were described as ‘pale’. The color perception of the panelists is in contrast to the measured color values of the fresh fillets, where the FM fillets were the brightest. Moreover, the redness level of the FM fillets was lower, and the yellowness level was slightly higher than in the other groups. This suggests the impression that either the yellowness level had a larger influence on the perception of the panelists or that further processes during preparation led to a serious change in the fillet color. This is very interesting, because the dE value of the fresh filets is low and the panelists’ description were not expected.

Lu et al. (2003) also showed that solely feeding spirulina to tilapia did not lead to sensorial differences [69]. Pike-perch and tilapia are both *Percomorphaceae* and seem to get along well with feeding on cyanobacteria regarding the sensory aspects of their fillets. However, it could be that in different countries, perception of fillet color is different. While some countries prefer more colored fillets, some prefer a more natural appearance.

#### 3.2.2. Rainbow Trout

The paired comparison tests of rainbow trout fillets (Figure 1, Table S2) showed more significant differences than the sensory evaluation of pike-perch. The frequency of noted comments was almost the same for both species (Table 5). In this comparison (Tests I–III), the attribute ‘too dark’ was the most noted one. The FM fillets showed significant differences in the intensity of color (Test II, expressed aspects) and were described as ‘too dark’ or ‘yellow to brown’. C fillets had the preferred color (Figure 1, Test II, Table S2), but were also described as ‘pale’ and ‘light’ compared to FM fillets and as ‘too dark’, ‘red’, ‘white’, and ‘pale’ compared to S fillets, whereas the S fillets’ descriptions were more inconsistent (‘yellow to red’, ‘pale’, and ‘brown’, Test III). The fresh FM fillets had the highest redness and yellowness levels. In the sensory evaluation, the FM fillets were described as ‘darker’ than the S and C fillets. These observations match with the previously determined color values. In the sensory evaluation, C fillets were described as ‘pale’, but a significant majority of the panelists preferred them against the S fillets.

The color values of the fresh C fillets also had lower redness and yellowness levels compared to the S fillets. This trend was also stated by Teimouri et al. (2013), because with increasing spirulina content in the feed, luminosity decreased, and the redness and yellowness levels increased [20]. Conversely, the control group also had the highest L value and the lowest redness and yellowness values here. The odors of all groups were balanced and did not show any significant differences concerning intensity and preference (Table 5). The most-noted attributes for the C fillets in Test III were from ‘no flavor’ to ‘too intense’ over ‘pungent’ or ‘musty’. There were no preferences for fillet texture. The C fillets showed a more intense texture in Test II but were not recognized in Test III. The FM fillets were described to be ‘too firm’ in Test I, but this trend was not identified in the other tests (Table 5).

Craft et al. (2016) explained that with a higher protein content, and therefore a lower fat content, fish fillets can become ‘too firm’ [65]. The significantly lower fat content of the FM fillets in the present study supports this statement, whereas the lower protein content contradicts this hypothesis. In Test I, FM fillets showed a more intense taste, but the preference in Test I was balanced. In Test II, control fillets had a more intense taste, but also here the preference was balanced. In Test III, the expressed tastes of S and C fillets were balanced, whereas the S fillets were preferred. Both fillet groups were described to have ‘a foreign taste’, but the C fillets were also described as ‘fishy’ and ‘too intense’. Typical off-flavors and odors such as ‘earthy’, ‘musty’, or ‘pungent’, also being noted in the present tests, can be assigned to metabolites that derive from microorganism-supplemented feeds [70,71] or can result from contaminations in the recirculation of the deep water system associated with possible accumulations of off-flavor compounds.

## 4. Conclusions

The present study showed that the substitution of fish meal and oil with a microorganism mix is not an optimal diet for pike-perch. Even though the present study period was longer than the one performed in a previous study [13], there was still not a well feed adaption of the pike-perch. The use of this new feed mix for pike-perch farming regarding the growth performance is not recommended at the moment. Promisingly, the sensorial differences between the fillets resulting from the standard and the feed mix were not significant. From this view, it can be a starting point to further optimize similar feed mixes for pike-perch farming.

Regarding the rainbow trout population, the present study showed that the growth performance and morphometric parameters of the feed mix groups reached a similar result as the conventionally fed groups. The growth of the FM groups was slightly reduced, but those deficits could be compensated, when the growing period is enlarged until reaching a marketable size. Consumers’ attitudes towards growth duration and individual size in fish farming through more sustainable feed should also be generally reconsidered, all being similar aspects to those associated with other sustainably grown livestock. The sensory aspects of the feed mix in rainbow trout farming seem to be positive for the consumer and promising for future studies. The numerous comments regarding the sensory impression should not be interpreted too negatively, as the comparison tests hardly showed any significant differences in preferences of the fillets.

This can be a point for the start of a larger consumer acceptance study.

In general, these observations show that low dE values can have a large effect on the final products. In this context, it is questionable whether traditional customers are willing to pay for a (white) fish with a conspicuous fillet color or whether they can be encouraged to buy those by additional information or advertising. The most important attribute for customers overall is still the taste. In this context, panelists’ statement of ‘foreign taste’ was balanced over all groups, and the panelists had no preference.

Further, it can be stated that in the present study, the partial substitution of fish meal and oil led to fish fillets without significant sensorial differences compared to fillets of fish that were fed with the standard diet. Occurrences of texture variants, color differences, off-flavors, and odors were minimal. Even when there were significant differences in the intensity of attributes, it did not have a great impact on the preference choice of the panelists.

## Figures and Tables

**Table 1:** Growth and feeding performance of pike-perch (*S. lucioperca*) and rainbow trout (*O. mykiss*).

Feeding Trial Parameter	Pike-Perch Standard Industrial			Pike-Perch FEED Mix			Pike-Perch Control			Rainbow Trout Standard Industrial			Rainbow Trout Feed Mix			Rainbow Trout Control		
Weight _initial_ (g)	186	±	41	171	±	42	169	±	48	126	±	26	144	±	38	148	±	30
Weight _end_ (g)	452	±	103 ^FK^	357	±	74	392	±	67 ^K^	508	±	117 ^F^	409	±	109	530	±	118 ^F^
Weight gain (g)	278	±	103 ^FK^	184	±	74	217	±	67 ^K^	382	±	117 ^F^	265	±	109	381	±	118 ^F^
Weight gain (%)	243			209			232			403			243			358		
Total length _end_ (cm)	35.5	±	3.0	35.0	±	2.0	35.3	±	1.8	33.8	±	2.5	32.4	±	2.5	34.9	±	2.2 ^F^
FCR	1.14 ^F^			1.57			1.05 ^F^			0.98			1.03			0.93		
SGR (% day^−1^)	0.78	±	0.21 ^F^	0.58	±	0.17	0.66	±	0.14	1.18	±	0.26 ^F^	0.88	±	0.23	1.09	±	0.20 ^F^
Survival rate (%)	99.3			99.2			98.6			94.7			93.8			92.3		
k-factor	1.00	±	0.12 ^FK^	0.83	±	0.07	0.88	±	0.06 ^K^	1.29	±	0.09 ^F^	1.18	±	0.11	1.23	±	0.11
HSI (%)	1.20	±	0.21	1.20	±	0.19	1.08	±	0.18	1.79	±	0.29	1.89	±	0.28	1.78	±	0.39
IFSI (%)	6.26	±	0.97 ^FK^	3.42	±	0.81	4.54	±	1.02 ^KF^	2.70	±	0.86 ^F^	1.96	±	0.70	2.29	±	0.58 ^K^

Significance levels: *p* < 0.05: ^F^ compared with feed mix, ^K^ compared within control and standard; HSI: hepatosomatic index; IFSI: intestine fat somatic index; SGR: specific growth rate.

**Table 2:** Fresh fillet and fresh liver color of pike-perch (S. lucioperca) and rainbow trout (O. mykiss) after 16 weeks.

Feeding Trial Parameter	Pike-Perch Standard Industrial			Pike-Perch Feed Mix			Pike-Perch Control			Rainbow Trout Standard Industrial			Rainbow Trout Feed Mix			Rainbow Trout Control		
Fillet color																		
L	42.4	±	2.1 ^F^	44.7	±	1.5	43.6	±	1.6	42.9	±	2.0 ^F^	41.2	±	1.6	42.9	±	2.7 ^F^
a	1.32	±	0.45	1.08	±	0.38	1.35	±	0.62	2.03	±	0.96	2.55	±	1.18	1.47	±	1.17 ^F^
b	−2.12	±	1.38	−1.58	±	0.87	−2.24	±	1.15	3.01	±	1.13 ^FK^	6.94	±	1.18	0.63	±	0.73 ^FK^
dE_Feedmix_	2.55	±	0.63 ^F^		−		1.53	±	0.31 ^F^	4.33	±	0.51 ^F^		−		6.64	±	1.21 ^F^
Liver color																		
L	47.1	±	5.0 ^F^	42.1	±	5.5	44.4	±	5.6	29.8	±	4.0 ^K^	28.7	±	3.6	26.4	±	4.5 ^K^
a	18.7	±	2.5	18.6	±	3.2	18.7	±	2.6	15.2	±	2.1	15.7	±	2.0	15.4	±	1.8
b	8.58	±	2.60	6.78	±	2.47	7.60	±	2.68	1.71	±	3.34 ^K^	1.30	±	3.17	−1.82	±	3.34 ^FK^
dE_week4_	7.45	±	2.42	5.81	±	3.81	6.75	±	3.90	4.78	±	2.97 ^K^	4.69	±	2.20	6.79	±	3.34 ^FK^
dE_Feedmix_	4.95	±	0.91 ^F^		−		2.08	±	0.62 ^F^	1.24	±	0.43 ^F^		−		3.91	±	0.91 ^F^

L = luminosity; a = redness level; b = yellowness level; d_Eweek4_ = color difference to Week 4; dE_feedmix_ = color difference to feed mix week 16; Significance levels: *p* < 0.05: ^F^ compared with feed mix fillets, ^K^ compared within standard and control feed.

**Table 3:** Nutritional parameters and lipid quality parameters of rainbow trout (*O. mykiss*) [31] and pike-perch (*S. lucioperca*) after 16 weeks of feeding.

Feeding TrialParameter [% Biomass]	Pike-Perch Standard Industrial			Pike-Perch Feed Mix			Pike-Perch Control			Rainbow Trout Standard Industrial			Rainbow Trout Feed Mix			Rainbow Trout Control		
Dry Mass	23.9	±	0.6 ^F^	23.6	±	0.6	24.4	±	0.6 ^F^	27.63	±	0.69	28.01	±	0.69	28.14	±	0.68
Ash	1.2	±	0.03	1.2	±	0.03	1.2	±	0.03	1.3	±	0.1	1.3	±	0.1	1.3	±	0.1
Proteins	21.2	±	0.53 ^F^	20.5	±	0.51	20.3	±	0.51 ^F^	19.4	±	0.5	19.1	±	0.5	19.4	±	0.5
Fat	1.1	±	0.03	1.1	±	0.03	1.3	±	0.03	8.2	±	0.2 ^F^	7.5	±	0.2	7.8	±	0.2 ^F^
Selected Fatty Acids[g fatty acid 100 g^−1^ biomass]																		
C16:0 palmitic acid	0.26	±	0.01	0.32	±	0.01	0.30	±	0.01	1.42	±	0.04	1.46	±	0.04	1.39	±	0.30
C16:1 palmitoleic acid	0.01	±	0.01 ^F^	0.07	±	0.01	0.01	±	0.01 ^F^	0.04	±	0.01	0.05	±	0.01	0.04	±	0.01
C18:0 stearic acid	0.08	±	0.01	0.08	±	0.01	0.08	±	0.01	0.42	±	0.01	0.43	±	0.4	0.40	±	0.01
C18:1 oleic acid	0.38	±	0.01	0.33	±	0.01	0.41	±	0.01	0.30	±	0.01	0.25	±	0.29	0.29	±	0.01
C18:2 linoleic acid (LA)	0.10	±	0.01	0.10	±	0.01	0.10	±	0.01	0.78	±	0.01	0.65	±	0.01	0.76	±	0.02
C18:3 y-linolenic acid	<0.005			<0.005			<0.005			0.01	±	0.01	0.01	±	0.01	0.02	±	0.01
C18:3 α-linolenic acid (ALA)	0.02	±	0.01 ^F^	0.01	±	0.01	0.02	±	0.01 ^F^	0.14	±	0.01 ^F^	0.10	±	0.01	0.15	±	0.01 ^F^
C20:4 arachidonic acid	0.01	±	0.01 ^F^	<0.005			0.01	±	0.01	0.02	±	0.01	0.02	±	0.01	0.03	±	0.01
C20:5 eicosapentaenoic acid (EPA)	0.03	±	0.01 ^F^	0.01	±	0.01	0.03	±	0.01 ^F^	0.06	±	0.01	0.04	±	0.01	0.11	±	0.01 ^F^
C22:5 docosapentaenoic acid	<0.005			<0.005			<0.005			0.01	±	0.01	0.02	±	0.01	0.03	±	0.01
C22:6 docosahexaenoic acid (DHA)	0.05	±	0.01 ^F^	0.03	±	0.01	0.06	±	0.01 ^F^	0.11	±	0.01	0.15	±	0.01	0.26	±	0.01 ^F^

Significance levels: *p* < 0.05: ^F^ compared with feed mix fillets, ^K^ compared within standard and control feed; LOQ = 0.005% biomass.

**Table 4:** Panelists’ commentary/impressions and frequency of occurrence regarding the paired comparison tests of pike-perch. The numbers describe how often the respective attribute is mentioned by the panelists.

	Test I				Test II				Test III			
Sensory Attribute	S		FM		C		FM		S		C	
Odor	foreign smell	3	foreign smell	2	soft	1	foreign smell	2	foreign smell	1	foreign smell	3
	herbal	1										
	intense	1	intense	3			intense	1				
					no aroma	3	no aroma	2				
							pungent	1	pungent	1	pungent	2
					musty	1	musty	1				
							not intense	1			not intense	2
					fishy	1					fishy	1
									sweet	1		
Color			darker	1	darker	1	darker	1				
			gray	4			gray	5				
	pale	1			pale	6	pale	1	pale	3	pale	3
					dry	1						
					fibrous	1						
Texture	too soft	1			soft	2	too soft	1	too soft	2	too soft	1
	firm	1			firm	1	firm	3	too firm	1	too firm	5
					voluminous	1						
Taste	foreign taste	3	foreign taste	2	foreign taste	1			foreign taste	1	foreign taste	1
	raw potato	4	raw potato	2	raw potato	2	raw potato	1			raw potato	4
	bland	1			bland	3			bland	1	bland	2
	sweet	1			sweet	1			sweet	1		
					musty	1	musty	2			musty	1
									bitter	1	bitter	1
									not intense	1	not intense	2
											fishy	2
											dry	1

S = Standard Industrial Diet; FM = Feed Mix; C = Control.

**Table 5:** Panelist’s commentary/impressions and frequency of occurrence regarding the paired comparison tests of rainbow trout. The numbers describe how often the respective attribute is mentioned by the panelists.

	Test I				Test II				Test III			
Sensory Attribute	S		FM		C		FM		S		C	
Odor			pungent	1	pungent	1	pungent	1	pungent	1	pungent	2
	fishy	2	fishy	3	fishy	1			fishy	1		
									sweet	2	sweet	2
			musty	1			musty	1	musty	1	musty	1
									foreign smell	1	foreign smell	1
									not aroma	4	no aroma	2
											not intense	1
											too intense	1
Color			yellow	1			yellow	1	yellow to red	2	red	2
			too dark	3			too dark	4			too dark	5
					pale	1			pale	1	pale	2
					light	1					white	2
							brown	1	brown	1		
Texture	too soft	1			too soft	1	too soft	1	too soft	2		
	too firm	2	too firm	4	too firm	1					too firm	1
					dry	1	dry	1				
	juicy	1										
Taste	bland	2	bland	5	bland	1						
	musty	1	musty	1							musty	1
	raw potato	1							raw potato	1		
	bitter	2	bitter	2								
	foreign taste	2	foreign taste	1	foreign taste	1	foreign taste	1	foreign taste	2	foreign taste	2
			fishy	2	fishy	4			fishy	1	fishy	3
			aquaous	1					aquaous	2		
									balanced taste	2	balanced taste	1
											smooth	1
											great taste	1
			not intense	1							not intense	1
			intense taste	1							too intense	2
			fatty	1								

S = Standard Industrial; FM = Feed Mix; C = Control.

## Data Availability

The data sets presented in this study are available on request from the corresponding author.

## Supplementary Materials

The following are available online at https://www.mdpi.com/article/10.3390/foods10081799/s1, Table S1. Paired comparison tests of pike-perch. The panelists decided which of the tested group has the more expressed attribute and which attributes of the groups they prefer. If the panelists had the impression there were no difference, the answer ND was given; Table S2. Paired comparison tests of rainbow trout. The panelists decided which of the tested group has the more expressed attribute and which attributes of the groups they prefer. If the panelists had the impression there were no difference, the answer ND was given.
