# Supplementary material for: Pike-Perch (Sander lucioperca) and Rainbow Trout (Oncorhynchus mykiss) Fed with an Alternative Microorganism Mix for Reducing Fish Meal and Oil—Fishes’ Growth Performances and Quality Traits
Source: Foods. 2021 Aug 4;10(8):1799. doi: 10.3390/foods10081799 (PMC8394391; doi:10.3390/foods10081799)
Supplement: Supplementary file 1 [file foods-10-01799-s001.zip › foods-1270226-supplementary.pdf]

Table S1. Paired comparison tests of pike-perch. The panelists decided which of the tested group has the more expressed attribute and which attributes of the groups they prefer. If the panelists had the impression there were no difference, the answer ND was given.

Attribute	Test I			Test II			Test III		
	**Standard Industrial**	**ND**	**Feed Mix**	**Control**	**ND**	**Feed Mix**	**Standard Industrial**	**ND**	**Control**
Expressed									
Odor	6	7	11	5	8	11	3	5	16*
Color	2	14	9	8	9	7	5	14	5
Texture	6	6	12	5	4	15	2	13	9
Taste	9	8	8	7	9	8	6	7	11
Preferred									
Odor	5	10	9	8	8	8	10	6	8
Color	5	17	1	11	10	3	5	15	4
Texture	8	10	6	9	6	9	7	14	3
Taste	4	10	9	7	9	8	8	10	6

Significance levels: * $p < 0.20$; ** $p < 0.05$; ND = no differences.

Table S2. Paired comparison tests of rainbow trout. The panelists decided which of the tested group has the more expressed attribute and which attributes of the groups they prefer. If the panelists had the impression there were no difference, the answer ND was given.

Attribute	Test I			Test II			Test III		
	**Standard Industrial**	**ND**	**Feed Mix**	**Control**	**ND**	**Feed Mix**	**Standard Industrial**	**ND**	**Control**
Expressed									
Odor	9	7	8	9	10	5	5	6	13
Color	3	14	7	4	0	20**	18**	4	2
Texture	7	7	10	16*	6	2	8	8	8
Taste	6	4	14	14	4	6	9	5	10
Preferred									
Odor	12	7	5	4	13	7	9	7	8
Color	8	15	1	13	4	7	0	5	19**
Texture	11	8	5	6	11	7	4	13	7
Taste	12	3	9	9	5	10	14	5	5

Significant levels: * $p < 0.20$; ** $p < 0.05$; ND = no differences.
